# Use of Digital Droplet PCR to Detect *Mycobacterium tuberculosis* DNA in Whole Blood-Derived DNA Samples from Patients with Pulmonary and Extrapulmonary Tuberculosis

**DOI:** 10.3389/fcimb.2017.00369

**Published:** 2017-08-11

**Authors:** Jiaru Yang, Xinlin Han, Aihua Liu, Xiyuan Bai, Cuiping Xu, Fukai Bao, Shi Feng, Lvyan Tao, Mingbiao Ma, Yun Peng

**Affiliations:** ^1^Yunnan Key Laboratory for Tropical Infectious Diseases Kunming, China; ^2^Yunnan Collaborative Innovation Center for Public Health and Disease Control Kunming, China; ^3^Institute for Tropical Medicine, Kunming Medical University Kunming, China; ^4^Department of Biochemistry and Molecular Biology, School of Basic Medical Science, Kunming Medical University Kunming, China; ^5^Yunnan Province Base for International Scientific and Technological Cooperation in Tropical Diseases Kunming, China; ^6^Department of Microbiology and Immunology, School of Basic Medical Science, Kunming Medical University Kunming, China; ^7^Departments of Medicine and Academic Affairs, National Jewish Health Denver, CO, United States

**Keywords:** Droplet Digital PCR, *Mycobacterium tuberculosis*, tuberculosis, molecular diagnosis

## Abstract

Tuberculosis (TB) is a chronic infectious disease that has been threatening public health for many centuries. The clinical diagnostic procedure for TB is time-consuming and laborious. In the last 20 years, real-time fluorescence-based quantitative PCR (real-time PCR) has become a better alternative for TB diagnosis in clinics due to its sensitivity and specificity. Recently, digital droplet PCR (ddPCR) has been developed, and it might be an ideal alternative to conventional real-time PCR for microorganism detection. In this study, we aimed to assess the capacity of ddPCR and real-time PCR for detecting low levels of circulating *Mycobacterium tuberculosis* (MTB) DNA. The study involved testing whole blood samples for an MTB DNA target (known as IS*6110*). Blood samples were obtained from 28 patients with pulmonary TB, 28 patients with extrapulmonary TB, and 28 healthy individuals. The results show that ddPCR could be used to measure low levels of MTB DNA, and it has the potential to be used to diagnose pulmonary and extrapulmonary TB based on clinical samples.

## Introduction

Tuberculosis (TB), which is caused by *Mycobacterium tuberculosis* (MTB) infection, is a chronic infectious disease that has been threatening public health for many years. Approximately one third of the world's population is infected with MTB (Tomioka et al., 2011). About 8 million new infections and 2 million TB-causing deaths occur annually (Baumann et al., 2006). In 2013, there were an estimated 9 million cases and 1.5 million deaths worldwide. Early and accurate detection of TB, as a global priority for TB control, will ensure that more TB cases are correctly diagnosed and treated (World Health Organization, 2015).

However, there is no gold standard for the diagnosis of latent TB infection (Wang et al., 2013), which means that some patients with TB do not have a chance of being appropriately treated and cured. Recent studies have shown that 20% of TB cases are extrapulmonary forms, including TB affecting the lymph nodes, pleura, and osteoarticular areas. This remains especially challenging for diagnosis because of the small amount of MTB present at the sites of the disease and the difficulty of obtaining clinical specimens from deep-seated organs (Golden and Vikram, 2005; Noussair and Bert, 2009).

Thus, nucleic acid amplification tests are increasingly used for the rapid detection of TB, especially real-time fluorescence-based quantitative PCR (real-time PCR), as it can be a sensitive and fast diagnostic method (Broccolo et al., 2003; Theron et al., 2014). Real-time PCR results are also used as a valuable confirmatory factor when deciding whether presumptive anti- TB treatment should be continued or quitted. Therefore, the use of real-time PCR is conducive to decreasing costs and potential toxicity (Noussair and Bert, 2009; Theron et al., 2014). An emerging technology known as digital droplet PCR (ddPCR) is a potential alternative to conventional real-time PCR for microorganism detection (Boizeau et al., 2014).

This new technology can detect the copy numbers of target DNA in small volumes of nucleic acid without requiring a calibration curve (Sedlak et al., 2014). In comparison to real-time PCR, this new technology partitions the initial PCR mixture into 20,000 microdroplets, which undergo PCR independently. The microdroplets that contain target DNA are labeled “positive” microdroplets and the rest are labeled “negative” microdroplets. After the PCR procedure has finished, the fluorescence signal of every microdroplet is detected. According to the fluorescence intensity of every microdroplet, a computer can be used to assess the copy number of the target DNA in the original DNA sample using Poisson statistics (based on the ratio of positive to total partitions; Huang et al., 2015). The advantages of ddPCR include the fact that the whole TB diagnostic procedure is potentially more accurate and highly sensitive, which makes this new technology a potential alternative for use in the clinical diagnosis of TB.

In this study, we used ddPCR to detect an MTB DNA target (known as IS*6110*) in whole blood samples from 28 patients with pulmonary TB, 28 patients with extrapulmonary TB, and 28 healthy individuals. The IS*6110* (GeneBank no. X52471) is a specific MTB DNA insert sequence which was found in 1990. Currently, it is regarded as an ideal target sequence of MTB nuclear acid diagnosis (Mcadam et al., 1990; Thierry et al., 1990a,b; Rao et al., 2009; Albuquerque et al., 2014; Hwang et al., 2015). We aimed to assess the capacity of ddPCR, in comparison to real-time PCR, for detecting very low levels of circulating MTB DNA. To our knowledge, this is first report on the diagnosis of TB in clinics using ddPCR with whole blood samples from patients.

## Materials and methods

### Subjects and sample collection

#### Subjects

The diagnosis of lung or extrapulmonary TB was clinically confirmed in accordance with National Institute for Health and Care Excellence (NICE) Guideline 33 on Tuberculosis (2006 and 2016 versions, UK) and the Chinese Clinical Diagnosis and Treatment Guidelines: Tuberculosis (2005, China). The confirmed diagnoses were based on a systematical analysis and integration of patients' medical history, current clinical symptoms, physical signs, imaging technologies (X-ray, computed tomography [CT], and positron emission tomography [PET]), molecular diagnostics (PCR and qPCR), microbiological examinations (BD Bactec MGIT 960 bacterial culture for samples with different resources and specimen smear staining), and immunological methods (interferon-gamma release assays and/or tuberculin skin tests). According to the WHO TB case definition criteria, the patients included in our study were divided into two groups: new cases of TB and cases of TB involving retreatment (Albuquerque et al., 2014). The general patient information of the patients included in our study is summarized in Table 1.

**Table 1:** General information from all the patients with TB.

**Baseline variable**	**Number of TB patients (%)**
**SEX**	
Male	35 (62.5)
Female	21 (37.5)
**AGE**	
<20	6 (10.71)
20–39	22 (39.29)
40–60	21 (37.5)
>60	7 (12.5)
**TB CLASSES**	
New case of TB	44 (78.57)
Case of TB involving retreatment	12 (21.43)
**TB TYPES**	
Pulmonary TB	28 (50)
Extrapulmonary TB	28 (50)
(1) Tubercular meningitis	5 (8.92)
(2) Abdominal TB	4 (7.14)
(3) Tuberculous pleurisy	3 (5.36)
(4) Tuberculous lymphadenitis of the neck	3 (5.36)
(5) Bone TB	12 (21.43)
(6) Pelvic TB	1 (1.78)

The study was approved by the local ethics committee of Kunming Medical University, Kunming, People's Republic of China, and signed informed consent forms were obtained from each study participant. The methods set out in this article were carried out in accordance with the relevant guidelines of Kunming Medical University.

Our study didn't include any vulnerable populations, so this part is not applicable.

#### Sample collection

Whole blood samples were collected from patients prior to the treatment with anti-TB drugs. Samples were collected from 28 patients with active pulmonary TB and 28 patients with extrapulmonary TB, and the anti-coagulant ethylene diamine tetraacetic acid (EDTA) was added. In addition, for patients with a clinically confirmed extrapulmonary TB diagnosis, lesion tissue samples were collected from the bone and spine during surgical operations at the Third Hospital of Kunming and Third Affiliated Hospital of Kunming Medical University, cultivated in BD Bactec MGIT 960 System and DNA-extracted for PCR to reach to a microbiologically confirmed extrapulmonary TB diagnosis. Whole blood samples were also collected from 28 healthy individuals at Kunming Blood Center, and the anti-coagulant EDTA was added. The whole blood samples were quickly stored at −80°C until DNA extraction.

### DNA extraction

A total of 85 DNA samples were extracted from the whole blood samples, which comprised 56 patients with different kinds of TB and 28 healthy individuals (as the healthy control group). In addition, for the MTB-positive control group (*n* = 3), three DNA samples were extracted from three lesion tissue samples of three patients with bone TB. For the non-MTB DNA control group (*n* = 3), DNA samples were obtained from human THP-1 cells.

The total DNA extraction from the whole blood samples involved the following procedures: (1) First, 200 μL whole blood was mixed with 800 μL Trizol and left to stand for 5 min. Subsequently, 300 μL chloroform was added to the mixture, which was fully mixed and left to stand for 5 min. Next, the mixture was centrifuged at 12,000 rpm for 5 min. The middle layer of the mixture was collected for use in the next step after the supernatant had been discarded. (2) Subsequently, 1 mL sodium citrate (0.1 M) in 10% ethanol and 4 μL RNaseA (100 mg/mL) was added, and the solution was fully mixed and left to stand for 5 min. Finally, the mixture was centrifuged at 12,000 rpm and the supernatant was discarded. This procedure was repeated, without adding RNaseA in the second round. (3) The precipitate was then fully mixed with 800 μL phenol/chloroform/isoamyl alcohol (25:24:1) and centrifuged at 12,000 rpm for 5 min. The middle layer of the mixture was collected. This step was then repeated. (4) Anhydrous ethanol was added to the mixture and fully mixed to allow DNA precipitation. The mixture was centrifuged at 12,000 rpm for 5 min and the supernatant was discarded. This step was then repeated. (5) precipitated DNA was dissolved in 10 μL Tris-EDTA buffer solution and kept −80°C for use.

The non-MTB DNA samples were extracted from human THP-1 cells using the above-mentioned DNA extraction protocol.

### Detection of the MTB-specific IS*6110* sequence using ddPCR

The ddPCR procedure was conducted using a QX100 Droplet Digital PCR System (Bio-Rad, California, USA). The reaction was performed using 2 × QX200 ddPCR EvaGreen Supermix (Bio-Rad, California, USA), according to the manufacturer's protocol. A no-template control and non-MTB DNA control were used in every ddPCR batch. The total volume used in each ddPCR procedure was 20 μl. The sequences of specific primers used to amplify the MTB-specific IS*6110* sequence (Genebank ID: X52471) were as follows: forward: 5′-ACCGAAGAATCCGCTGAGAT-3′ and reverse: 5′-GACGCGGTCTTTAAAATCGC-3′. The products were 83 bp in length. The ddPCR procedure was carried out under the following conditions: initial denaturation at 95°C for 5 min followed by 35 cycles of 15 s at 95.0°C, 30 s at 55.3°C, and 4°C at 5 min, and finally, 90°C at 5 min for signal stabilization. The quantification data were analyzed with QuantaSoft software version 1.7.4 (Bio-Rad, California, USA). The results were presented as IS*6110* copy number per microliter DNA sample.

### Detection of the MTB-specific IS*6110* sequence using TaqMan teal-time PCR

The real-time PCR procedure was conducted using the CFX Connect Real-Time PCR Detection System (Bio-Rad, California, USA). The reaction was performed using 2 × Probe qPCR Mix (TaKaRa, Dalian, China), according to the manufacturer's protocol. A no-template control and non-MTB DNA control were used in each PCR batch. The total volume used in each real-time PCR procedure was 25 μl. The sequences of specific primers used to amplify the MTB IS*6110* sequence (Genebank ID: X52471) were as follows: forward: 5′-ACCGAAGAATCCGCTGAGAT-3′, reverse: 5′-GACGCGGTCTTTAAAATCGC-3′, and probe: 5′-CGGGACAACGCCGAATTG-3′. The products were 83 bp in length. The real-time PCR was carried out under the following conditions: initial denaturation at 95°C for 30 s followed by 40 cycles of 5 s at 95.0°C and 30 s at 55.3°C. The quantification data were analyzed using CFX Manager version 3.1 (Bio-Rad, California, USA).

### Statistical analyses

Single-blind PCR reactions (i.e., where the experimenter was blind) were used to collect data for the comparative TB case vs. health control analysis. The results are expressed as means ± standard errors of the means (SEMs). The significance of the between-group differences in the mean IS*6110* copy number in DNA samples was evaluated using one-way analysis of variance (ANOVA) and the U test with Graph Pad Prism statistical software version 6.0. The differences were considered statistically significant at a value of *P* < 0.05. The difference between real-time PCR and ddPCR in terms of the ability to diagnose cases as positive for TB (pulmonary and extrapulmonary) was evaluated using the chi-square test with Graph Pad Prism version 6.0. Again, the differences were considered statistically significant at a value of *P* < 0.05.

## Results

### Specificity and sensitivity of ddPCR for the detection of MTB IS*6110*

**Figure 1:**
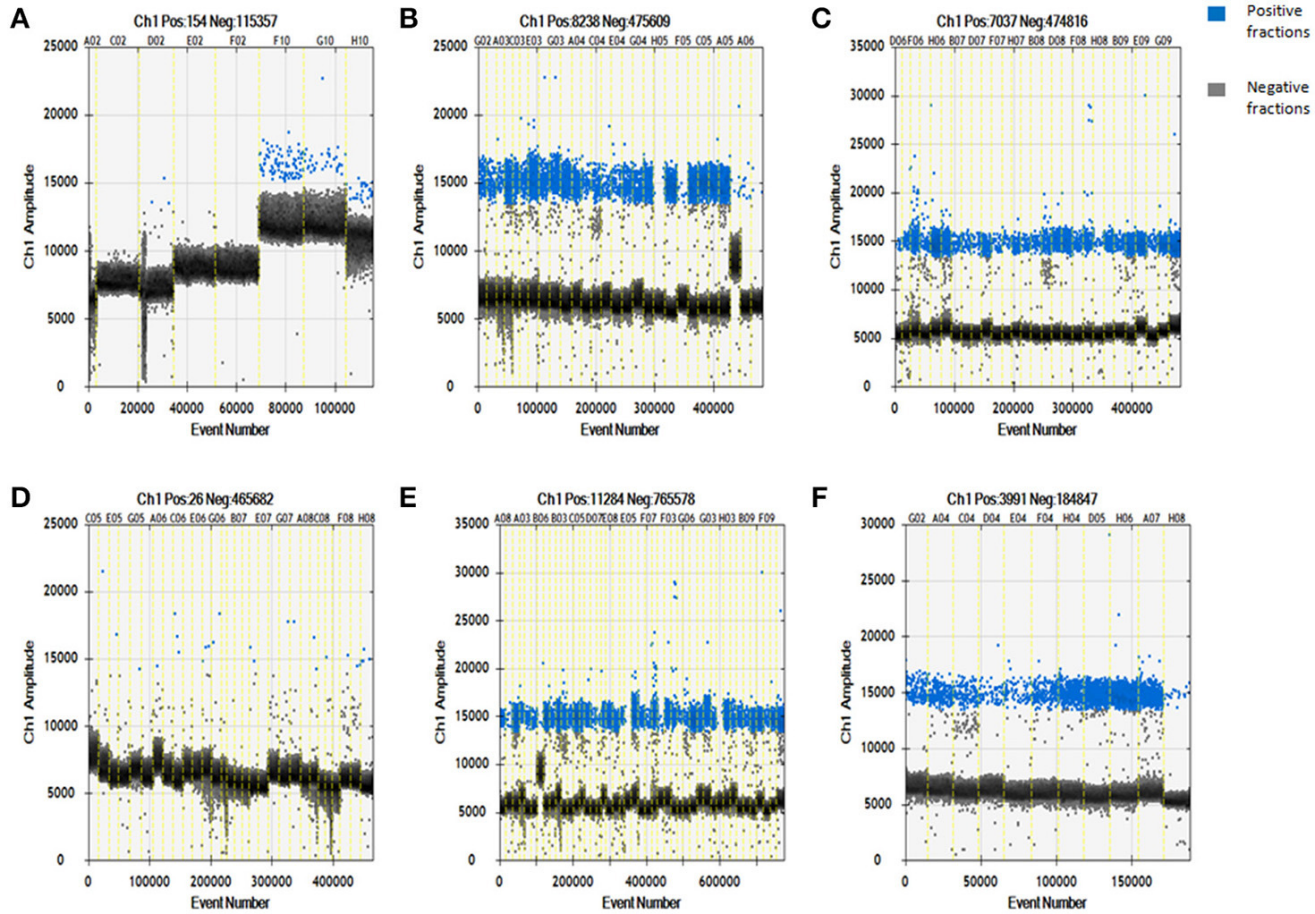
Specificity and sensitivity of digital droplet PCR for detecting *Mycobacterium tuberculosis*-specific target DNA in whole blood-derived DNA samples from patients with pulmonary or extrapulmonary TB. **(A)** Digital droplet PCR results for DNA samples in the template-free control, non-MTB DNA control, and *Mycobacterium tuberculosis* DNA-positive control groups. **(B)** Digital droplet PCR results for DNA samples from patients with pulmonary TB. **(C)** Digital droplet PCR results for DNA samples from patients with extrapulmonary TB. **(D)** Digital droplet PCR results for DNA samples from healthy controls. **(E)** Digital droplet PCR results for DNA samples from new cases of TB. **(F)** Digital droplet PCR results for DNA samples from cases of TB involving retreatment. The results are expressed as means ± standard errors of the means (SEMs) and presented as IS*6110* copy number per microliter DNA sample.

Using a QuantaSoft analysis, the IS*6110* copy number in each DNA sample was calculated with Poisson statistics (based on the ratio of positive to total partitions; Huang et al., 2015). As shown in Figure 1A, the IS*6110* copy number was undetectable in the no-template control (*n* = 1, sample serial number: C02); 0.63 ± 0.73 copies/μL in the non-MTB DNA control (*n* = 3, sample serial numbers: D02, E02, and F02); and 28.33 ± 4.29 copies/μL in the MTB-positive controls (*n* = 3, sample serial numbers: G10, H10, and F10). Thus, the lack of contamination during our study helps to ensure the reliability of the results and, according to the results for the MTB-positive controls, IS*6110* could be detected in the tissue infected with MTB.

The ddPCR results showed that a large number of IS*6110*-positive microdroplets were detected in the DNA samples from the patients with pulmonary TB, extrapulmonary TB, new cases of TB, and cases of TB involving retreatment, as shown in Figures 1B,C,E,F, respectively. There were also a few dispersed positive microdroplets in the healthy control group, as shown in Figure 1D. The non-MTB DNA control results indicated that nonspecific amplification probably caused the detection of very low levels of IS*6110* sequence in the healthy controls.

### IS*6110* copy number by TB type

The IS*6110* copy number in DNA samples from pulmonary TB patients (*n* = 28) was 201.80 ± 40.94copies/μL, and the copy number for extrapulmonary TB patients (*n* = 28) was 167.40 ± 40.82 copies/μL, as shown in Figures 1B,C, respectively. In addition, the IS*6110* copy number in DNA samples from healthy controls (*n* = 28) was 0.57 ± 0.14 copies/μL. In the healthy control group, we found that IS*6110* was undetectable in all samples.

### No differences in IS*6110* copy number in extrapulmonary TB patients with different infected organs

**Figure 2:**
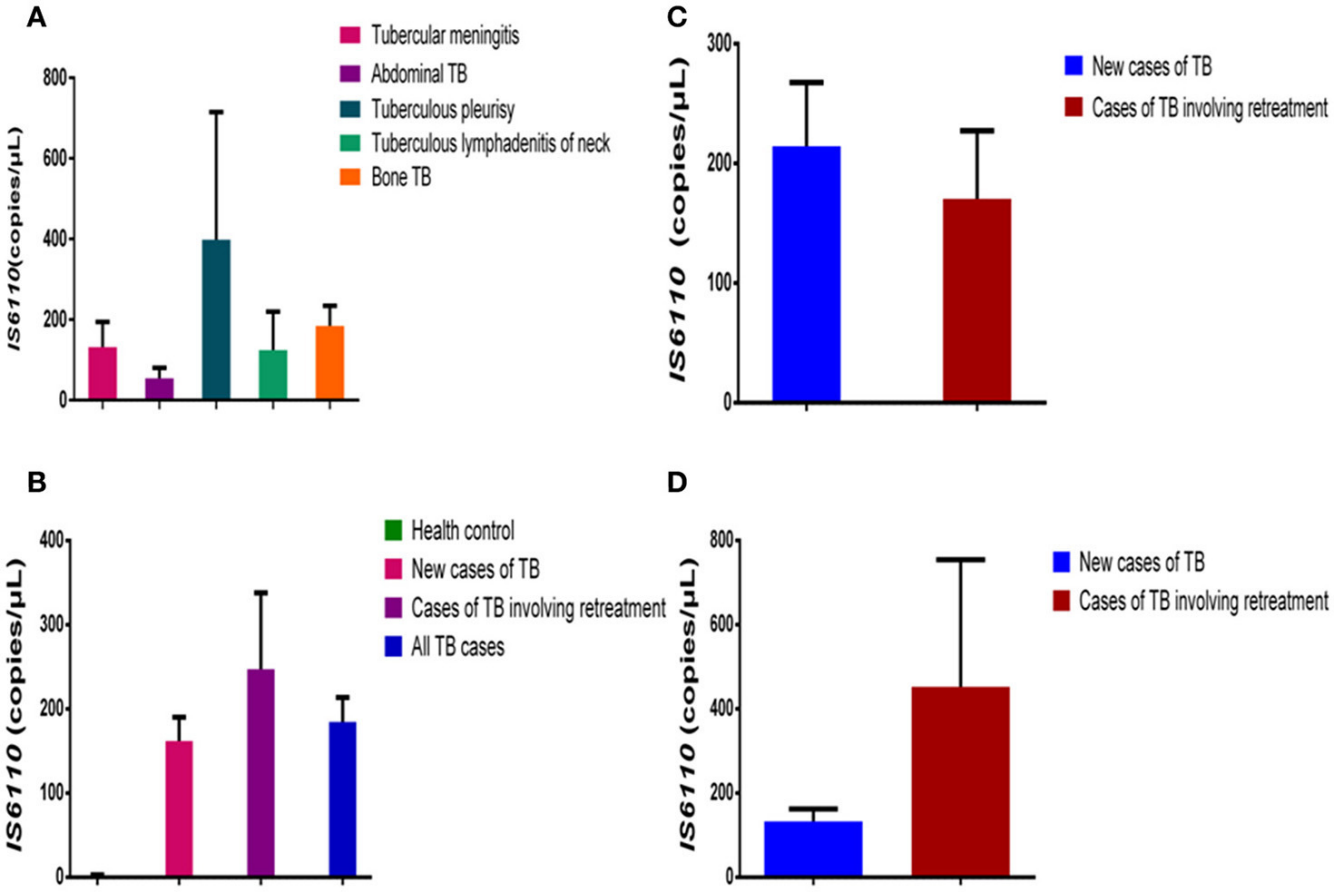
Comparison of IS*6110* copy number in DNA samples from different TB patient groups. **(A)** Mean IS*6110* copy number in DNA samples from extrapulmonary TB patients with different infected organs. **(B)** Mean IS*6110* copy number in DNA samples from healthy controls, new cases of TB, cases of TB involving retreatment, and all TB cases. **(C)** Mean IS*6110* copy number in DNA samples from new pulmonary TB cases and those involving retreatment. **(D)** Mean IS*6110* copy number in DNA samples from new extrapulmonary TB cases and those involving retreatment. The results are expressed as means ± standard errors of the means (SEMs) and presented as IS*6110* copy number per microliter DNA sample.

The patients with extrapulmonary TB who were enrolled in our study had six different kinds of TB: tubercular meningitis (131.40 ± 63.14 copies/μL, *n* = 5), abdominal TB (54.00 ± 26.55 copies/μL, *0* = 4), tuberculous pleurisy (398.00 ± 316.90 copies/μL, *n* = 3), tuberculous lymphadenitis of the neck (124.00 ± 95.52 copies/μL, *n* = 3), and bone TB (184.30 ± 50.24 copies/μl, *n* = 12). As shown in Figure 2A, there are no significant differences in the IS*6110* copy number in the DNA samples from the patients with different kinds of extrapulmonary TB.

### No differences in IS*6110* copy number between new cases of TB and cases of TB involving retreatment

The patients with TB were divided into two groups: new cases of TB (*n* = 44) and cases of TB involving retreatment (*n* = 12). The IS*6110* copy number in the DNA samples was 0.57 ± 0.14 copies/μL for the healthy controls, 162.10 ± 28.09copies/μL for the new cases of TB, 247.40 ± 90.52 copies/μL for the cases of TB involving retreatment, and 184.60 ± 29.09 copies/μL for all the TB cases together. In pairwise comparisons with the healthy controls, the IS*6110* copy number was significantly higher in the DNA samples from new cases of TB (*P* < 0.0001), cases of TB involving retreatment (*P* < 0.0001), and all the cases of TB (*P* < 0.0001) (Figure 2B). There was no significant difference between the new cases of TB, cases of TB involving retreatment, and total cases of TB (*P* > 0.05) (Figure 2B).

We further divided both the pulmonary and extrapulmonary TB cases into two subgroups each: new cases of TB and cases of TB involving retreatment. Regarding pulmonary TB, there was no significant difference between the new cases (214.40 ± 53.17 copies/μL, *n* = 20) and cases involving retreatment (170.40 ± 57.00 copies/μL, *n* = 8) (*P* > 0.05) (Figure 2C). Moreover, regarding extrapulmonary TB, there was also no significant difference between the cases involving retreatment (452.70 ± 301.50, *n* = 3) and the new cases (133.20 ± 28.96 copies/μL, *n* = 25) (*P* > 0.05) (Figure 2D).

### Comparison of ddPCR and real-time PCR for detecting pulmonary and extrapulmonary TB

The chi-square value for the comparison of the real-time PCR and ddPCR results for pulmonary TB detection was 18.67 (*P* < 0.0001) (Table 2) and the chi-square value for extrapulmonary TB detection was 16.93 (*P* < 0.0001) (Table 3). This indicates that ddPCR has advantages over real-time PCR for detecting low numbers of copies of MTB DNA in the peripheral blood of patients with pulmonary TB and extrapulmonary TB.

**Table 2:** Comparison of real-time PCR and ddPCR for the detection of pulmonary TB.

	**Pulmonary TB positive**	**Pulmonary TB negative**
ddPCR	28	0
Real-time PCR	14	14
Chi-square value = 18.67	*P* < 0.0001	α < 0.05

**Table 3:** Comparison of real-time PCR and ddPCR for the detection of extrapulmonary TB.

	**Extrapulmonary TB positive**	**Extrapulmonary TB negative**
ddPCR	28	0
Real-time PCR	15	13
Chi-square value = 16.93	*P* < 0.0001	α < 0.05

## Discussion

Effective prevention and treatment of TB requires early and less invasive TB diagnoses, highlighting the critical role of laboratories for rapidly and accurately detecting TB. Laboratory confirmation of TB is essential to ensure that individuals with TB are correctly diagnosed and acquire the appropriate treatment as soon as possible.

TB can be confirmed diagnostically when MTB is found in a clinical specimen taken from the patient and cultivated. Although, the results of other diagnostic methods may strongly suggest a TB diagnosis, they cannot be used to confirm a diagnosis.

A complete medical diagnosis of TB must include information from the patient's medical history, a physical examination, a chest X-ray, a microbiological examination (of sputum or some other appropriate sample), a nucleic acid amplification test, and sometimes, immunological tests (an interferon-gamma release assay and/or a tuberculin skin test), and occasionally an adenosine deaminase assay. The diagnosis may also include other scans (including X-rays) and surgical biopsies of the potentially infected sites.

For the clinical laboratory diagnosis of TB, selecting suitable sample types (which are typically used for TB diagnosis either based on nucleic acid testing or culturing) is critical. As MTB can infect many types of tissues and organs (which lead to different lesions, symptoms, and signs), the collection of the correct sample types relies on selecting the correct TB sites. For example, for pulmonary TB, the sample is typically sputum, and for extrapulmonary TB, common samples are pleural ascites fluid, gastric aspirate, lymph node aspirate, urine, blood, and even stool. However, in many cases, MTB may not enter the sputum (e.g., to allow the diagnosis of lung TB close to the airways) or the above-mentioned fluids (e.g., to allow the diagnosis of bone or brain TB). In general, MTB is present only at low concentrations in blood. However, as new highly sensitive techniques are being developed (such as fluid biopsy), it is possible to find low levels of MTB DNA in the blood.

Nucleic acid amplification tests are promising techniques for the rapid and specific detection of MTB, and they can be used to detect low-copy pathogen nucleic acid sequences. As MTB cultivation is time-consuming and laborious, nucleic acid amplification tests may be a good alternative for MTB diagnosis.

Real-time PCR and a newer assay named the Xpert MTB/rifampin (RIF) assay (which is based on real-time PCR) have been widely used for the clinical diagnostic of MTB (Lawn and Zumla, 2012; Albuquerque et al., 2014). However, real-time PCR relies on a calibration curve to calculate the MTB DNA copy number, which results in a highly complicated diagnostic process that requires that researchers spend a large amount of time customizing MTB standards in order to develop the calibration curve. In addition, the construction of standard reference substances (such as MTB cultures, which are widely used as reference standards for validating PCR results) could increase the difficulty of carrying out real-time PCR and make the generation of false positives and negatives more likely (Yong-sheng Sun et al., 2011). It has been reported that the use of an inappropriate reference standard could impact on the accuracy of real-time PCR results, and therefore on the diagnosis of TB (especially of extrapulmonary TB), so the combined results of multiple tests should be analyzed (Enrico et al., 2012). As a result of these reasons, real-time PCR has several limitations when it is used for the clinical diagnosis of bacterial infections such as MTB. Thus, it is necessary to consider a new technology with advantages over real-time PCR, and we have found just such an emerging technology, ddPCR, which may be a potential alternative to real-time PCR.

Without requiring the preparation of reference standards and the construction of a standard curve, ddPCR measures the absolute quantities of target DNA by partitioning the nucleic acids into discrete water-in-oil microdroplets that are assessed individually (Pinheiro et al., 2011). It offers highly reproducible low-copy number detection and good analytical sensitivity (Sanders et al., 2011). We extracted DNA from peripheral whole blood samples collected from 28 patients with pulmonary TB and 28 patients with extrapulmonary TB, detected MTB IS*6110* using ddPCR and TaqMan real-time PCR, and compared these results from the results from 28 healthy individuals. Furthermore, the chi-square value and p value indicated that real-time PCR was less able to diagnose cases as TB positive (regardless of whether the TB was pulmonary or extrapulmonary). We found a very low number of microdroplets associated with nonspecific amplification, but this did not affect the overall results. However, if poor-quality DNA samples are detected by ddPCR, the use of specific probes would be recommended to avoid nonspecific amplification. When used to compare new cases of TB and cases of TB involving retreatment, there was no significant difference in the detection of MTB IS*6110*.

The results indicate that copy numbers can be accurately assessed to two decimal places, which means that ddPCR is highly resistant to the PCR reaction inhibitors such as phenol, ethanol, EDTA, SDS, and so on, with high accuracy and sensitivity. The results show that ddPCR has the capacity to detect low levels of MTB DNA and the potential to be used to diagnose pulmonary and extrapulmonary TB based on clinical blood samples. Although recently there have been no discussion about TB diagnosis using ddPCR, the future of this technology is promising.

Why did we choose IS*6110* as the target of detection? The different MTB sequence targets such as IS*6110*, the 16S rRNA gene, and the 65-kDa protein gene (Rv0440), and devR (Rv3133c), have been used for the real-time PCR-based diagnosis of several clinical types of extrapulmonary TB (Promod et al., 2012), but IS*6110*, which was used in our study, has long been used for the detection of MTB-specific DNA sequences as a sensitive and fast diagnostic target because of its multiple copies in the MTB genome, which are believed to lead to higher sensitivity (Nadine et al., 2004). In short, we have provided the preliminary results to prove that ddPCR is a promising molecular diagnostic tool to detect low levels of MTB DNA and possesses the potential to be used to diagnose pulmonary and extrapulmonary TB based on clinical blood samples, but more multicenter study and larger number of clinical samples will be required to further confirm its sensitivity, specificity, and dependability.

## Author contributions

FB was responsible for the integrity of the work as a whole. JY and XH contributed to the data analysis. XB and AL contributed to the study design, data acquisition, and data analysis. SF, CX, LT, MM, and YP contributed to the data analysis. All the authors contributed to the data interpretation and manuscript drafting and all approved the final version.

### Conflict of interest statement

The authors declare that the research was conducted in the absence of any commercial or financial relationships that could be construed as a potential conflict of interest.
